# Orchestrated Cytokines Mediated by Biologics in Psoriasis and Its Mechanisms of Action

**DOI:** 10.3390/biomedicines10020498

**Published:** 2022-02-20

**Authors:** Aina Akmal Mohd Noor, Maryam Azlan, Norhanani Mohd Redzwan

**Affiliations:** 1 Immunology Department, School of Medical Sciences, Universiti Sains Malaysia, Kubang Kerian 16150, Kelantan, Malaysia; ainaakmal@student.usm.my; 2 School of Health Sciences, Universiti Sains Malaysia, Kubang Kerian 16150, Kelantan, Malaysia; maryamazlan@usm.my

**Keywords:** psoriasis, biologics, inhibitors, cytokines, immune cells, autoimmune disease

## Abstract

Psoriasis is an autoimmune disease mediated by disturbed T cells and other immune cells, and is defined by deep-red, well-demarcated skin lesions. Due to its varied etiologies and indefinite standard pathogenesis, it is challenging to consider the right treatment exclusively for each psoriasis patient; thus, researchers yearn to seek even more precise treatments other than topical treatment and systemic therapy. Using biologics to target specific immune components, such as upregulated cytokines secreted by activated immune cells, is the most advanced therapy for psoriasis to date. By inhibiting the appropriate pro-inflammatory cytokines, cellular signaling can be altered and, thus, can inhibit further downstream inflammatory pathways. Herein, the roles of cytokines with their mechanisms of action in progressing psoriasis and how the usage of biologics alleviates cellular inflammation are discussed. In addition, other potential pro-inflammatory cytokines, with their mechanism of action, are presented herein. The authors hope that this gathered information may benefit future research in expanding the discovery of targeted psoriasis therapy.

## 1. Introduction

Psoriasis is a chronic autoimmune disease characterized by significant dermal lesions caused by disturbed immune cells [1]. As reported by the National Psoriasis Foundation [2], psoriasis is quite prevalent and affects approximately 125 million people, which constitutes 2–3% of the global population, with no sexual bias [3,4]. Psoriasis is often associated with genetic predisposing disease, in which one-third of the global cases were initiated during childhood rather than due to exogenous triggers, such as skin trauma and chemical irritants [5,6]. The most common clinical observation for psoriasis is the well-demarcated inflamed lesions which usually appear with red and/or white scaly patches that often have silver patches [7]. Apart from the physical burden, psoriasis often affects social well-being as well as causing emotional and psychological stress [7,8]. Generally, there are varying types of psoriasis, for instance, plaque psoriasis, guttate psoriasis, erythrodermic psoriasis, pustular psoriasis and others, such as palmoplantar, nail and hair psoriasis. All these types are the results of impaired immune cell connections, especially within the integumentary layers, and are caused by some triggers [9].

These interrupted immune components lead to turmoiled T cells signaling and eventually trigger the keratinocytes on the epidermis layers to become hyperproliferative. Psoriasis patients experience fast skin replacement on the affected area due to truncated keratinocytes within just three to five days, where normal skin replacement is about 28 days [10]. Most of the lesions are associated with a deep-red colorization that comes with visible blood capillaries and/or dense erythema patterns on the psoriatic skin [11]. This is due to the intensified neovascularization which occurs because of the highly promoted angiogenesis activity that acts via angiogenic mediators. These mediators aid in forming numerous cellular infiltrations at the lesioned site [10,12]. With all the unique clinical observations, the Psoriasis Area and Severity Index (PASI) scoring system was created to guide the standardization of psoriatic lesions, and comprises three main parameters: erythema, skin thickness and scale formation [13,14].

All conditions stated above are the outcomes of pathophysiology for all types of psoriasis. However, to date, the benchmark of psoriasis pathogenesis is yet to be discovered, since the current understanding relies on hypothesizes relating to multifactorial etiologies [15,16]. Mapping the complete circuit of the psoriasis cellular pathogenesis framework is, therefore, a significant technological challenge. Understanding the complete psoriasis pathogenesis would be of great benefit for establishing ultimate treatments, since the chronic nature of psoriasis often leads to future relapse, hence requiring a long-term treatment instead [6]. Nonetheless, psoriasis is, indeed, caused by the chaos of triggered immune cells and its cytokines, such as tumor necrosis factor (TNF)-α, interleukin (IL)-17, IL-22, IL-23, and granulocyte–macrophage colony-stimulating factor (GM-CFS) that flare up throughout the pathway [17]. It was found that due to this cytokine storm episode, psoriasis patients are vastly vulnerable to comorbid diseases, such as psoriasis arthritis, Crohn’s disease, malignancy and even cardiovascular disease, which are mostly life-threatening [18,19,20]. Hence, the highlighted point here is the uncontrolled production of cytokines in regard to psoriasis would lead to various immune cells signaling in a chain reaction fashion, which leads to hyperproliferative keratinocytes and potentially excessive antimicrobial peptides (AMP), such as LL-37 [21,22].

## 2. Pathogenesis of Psoriasis

The sustained inflammation that leads to uncontrolled keratinocyte proliferation and faulty differentiation are the hallmarks of psoriasis. Multiple triggers could be from exogenous sources, for instance, infection, skin trauma, smoking habits, drugs, infections and occupational hazards [5]. A strong familial hereditary association of psoriasis-susceptible loci *PSORS* is also a cause of severe psoriasis development which can be detected at an early age [23,24]. If the disease is considered to be acquired, it might be from certain intrinsic conditions, such as hypertension, diabetes mellitus and predisposing metabolic syndrome [5,25].

**Figure 1:**
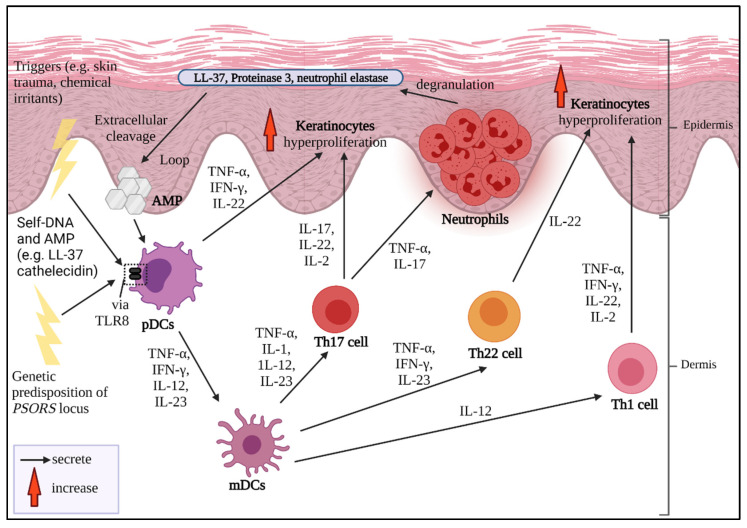
The summarized pathogenesis of psoriasis. Upon triggers, pDCs receive the signal via TLR8 and present the antigens to mDCs. mDCs activate Th1 and Th22 cells to release cytokines to generate hyperproliferative keratinocytes in the epidermis region. mDCs also present cytokines to Th17 cells to initiate keratinocyte hyperproliferation and the assembling of neutrophils to create Munro’s microabscesses. It is thought that once neutrophils undergo degranulation, this produces granular compounds, such as LL-37 and proteinase 3, which create an information loop to be detected again by pDCs. The cycle repeats. (AMP: antimicrobial proteins; *PSORS*: psoriasis susceptibility loci).

Indeed, the complete pathogenesis of psoriasis remains ambiguous, and the “Psoriatic Universe” is still indefinite and waiting to be fully explored [26]. The development of psoriasis can be divided into four main phases: triggered initiation, responsive innate immune response, stimulated adaptive immune response and excessive epidermal proliferation (Figure 1). It is highly suggested that nucleic acid complexes, such as AMP chains of cathelicidin LL-37 within the upper layer of the skin, stimulate plasmacytoid dendritic cells (pDCs) during the early onset of psoriasis. Plasmacytoid DCs produce cytokines such as IFN-γ, TNF-α, IL-12 and IL-23 to communicate with myeloid dendritic cells (mDCs). These cytokines then signal CD4^+^ and CD8^+^ T cells to undergo clonal expansion and produce IL-17 and IL-22. CD8^+^ T cells migrate and connect with the MHC I receptors of the keratinocytes to assemble chemoattractants and innate immune mediators [27]. Moreover, mDCs also stimulate the differentiation of T helper (Th)1, Th22 and Th17 cells. Th1 cells secrete IFN-γ, TNF-α and IL-2 which promotes the inflammatory factors by signaling keratinocytes and DCs. Conversely, Th22 cells produce IL-22, which is responsible for triggering keratinocyte-derived T cell-recruiting chemokines and gives rise to the changed dermal phenotype, comprising epidermal hyperplasia, acanthosis and parakeratosis. Th17 cells that are stimulated by IL-1, IL-23, IL-12 and TNF-α release IL-17 once they undergo migration towards the dermis [28,29]. The released IL-17 then signals the keratinocytes to express TNF-α and CC chemokine ligands (CCL20). The combination of IL-17 and TNF-α can assemble neutrophils and create Munro’s microabscesses. Neutrophils can undergo degranulation and produce granular compounds, such as neutrophil elastase (NE), proteinase 3, LL-37, reactive oxygen species (ROS), α-defensin with antimicrobial characteristics and lipocalin, as well as C-X-C-motif ligand (CXCL)8, IL-6 and CCL20 [30].

## 3. Psoriasis and Cytokines as Biologics Target

The currently available treatments for psoriasis are topical application, systemic therapy and biologics. Topical application is the only preferred treatment for mild psoriatic lesions and best acts on the surface of the lesioned sites in the short term. Hence, the epidermis layer in which keratinocytes become hyperproliferative can be softened and reduce the uncomfortable signs especially during flare-ups, which include itchy, tingling and burning sensations [5,31].

In tackling the recurrent flaring episodes, particularly in moderate to severe forms, some patients choose systemic therapy, especially when they are unable to respond well to topical application. However, the long-term administration of systemic therapy results in low acceptance and potential multiorgan failure, as well as probable skin carcinogenesis [5,32]. For example, methotrexate disturbs DNA synthesis, replication and reconstruction. Some systemic therapies, for instance, acitretin and cyclosporin, lead to the abnormality of liver function and subsequent dyslipidemia, hyperkalemia, hyperuricemia and hypomagnesemia [33].

Biologics are considered to be the most advanced treatment strategy with minimum risks. It is very beneficial for moderate-to-severe psoriasis patients, since this drug can selectively inhibit and/or dissolve targeted cytokines, hence reducing further inflammatory pathways [34,35]. Biologics are made of large and complex elements of combined monoclonal antibodies (mAb) with receptor fusion proteins which function to target immune mediators specifically [33]. They can specifically block any designated cytokines and their receptors from regulating the downstream signaling pathways [36].

The exploration of the usage of biologics has become worldwide after the discovery of the first successful introduction of alefacept, a type of biologic, in psoriasis [37]. This is the first instance of biologics in psoriasis using the dimeric fusion of the extracellular section of the human leukocyte function antigen-3 (LFA-3) and the Fc section of immunoglobulin (Ig) G1, which was approved by the US Food and Administration (FDA) in 2004 [38]. Alefacept is a non-cytokine-blocking agent, since it is designed to target the interaction of T cells by blocking LFA-3 signaling on CD2, thus deactivating the stimulation [38,39,40]. However, it is indicated that alefacept produces non-neutralizing antibodies [41] and side effects [42]. Hence, further research later yearned to focus more on cytokine inhibitors instead [42].

Cytokines play a massive role in orchestrating and assembling the immune cells in psoriasis; thus, this could be the most suitable target to be used as a promising treatment [18]. The discovery of an effective novel biologic gives new insights into how beneficial it is to target important immune components, such as cytokines, in alleviating psoriasis exasperation. Hence, it can support other advanced methods, such as the quantification of numerous cytokines receptors using both mRNA and cDNA microarrays [43,44]. Thus, it is crucial to inhibit cytokine signaling if that is the attempted exit strategy for psoriasis. Herein, the information of the main cytokine inhibition targets, along with potential cytokines and their mechanism of action in psoriasis, are discussed. A literature search was conducted in PubMed, Scopus, ScienceDirect and Google Scholar using the search terms “psoriasis” AND “biologics” AND (“cytokines” OR “treatment” OR “pathogenesis” OR “pro-inflammatory” OR “topical” OR “systematic” OR “inhibitors” OR “TNF-α” OR “infliximab” OR “etanercept” OR “adalimumab” OR “certolizumab” OR “pegol” OR “IL-17” OR “secukinumab” OR “ixekizumab” OR “brodalumab” OR “IL-23” OR “tildrakizumab” OR “guselkumab” OR “risankizumab” OR “IL-12/23” OR “ustekinumab” OR “IFN” or “interferon” OR “IL-1” OR “IL-36” OR “IL-6” OR “IL-8” OR “IL-21” OR “IL-17/23” OR “IL-22”).

## 4. Main Potential Cytokine Targets in Psoriasis

Herein, the profiles of the main cytokines that play a major role in psoriasis are summarized and discussed.

### 4.1. TNF-α Inhibitors

TNF-α is a proinflammatory cytokine secreted by DCs, T cells, macrophages and non-immune cells, such as fibroblasts; therefore, its selection in the revolution of inhibitor drugs is promising [44]. TNF-α acts as the main mediator during the initiation phase of classical psoriasis and can sustain the disease over the long term [45,46,47]. It interposes inflammatory cascades, promotes cell growth, neovascularization and apoptosis and regulates the aggregation of other immune cells to the site of the lesions [12]. TNF-α plays an important role in suppressing the regulatory T cells, preventing them from allowing the subsequent hyperproliferation of pathogenic T cells and IL-17-producing cells. As a result, IL-17 is unable to stimulate CD8^+^ T cells; instead, it is downregulated [48,49]. Hence, the serum level of TNF-α in psoriatic patients is highly elevated with a positive correlation with the PASI score. Since TNF-α is also considered to be the central cytokine in this autoimmune disease, it can give predictions of the exacerbation susceptibility of psoriasis. Psoriasis can, indeed, be inherited and run down the family tree; hence, by tracing *TNFA* genes on the short arm of chromosome 6, which comprises −238G > A, −380G > A and −875C > T, genetic polymorphism as the predisposing factor could give early insights and help to identify drug targets [50,51]. Studies have reported that these polymorphisms contribute to more than 50% of prevalence cases in psoriasis [43,44,52,53]. Moreover, TNF-α, as the homotrimer cytokine, is associated with altering the cell cycle, especially in keratinocytes and hair follicles in psoriasis [54]. Collectively, these findings suggest that TNF-α is, indeed, a good candidate with which to establish a specific treatment regime for psoriasis.

During the early onset of psoriasis, DCs release TNF-α along with other cytokines, such as IL-23, to signal for the assembling of CD4^+^ and CD8^+^ T cells. Eventually, T cells migrate to the upper layer of the skin, which is near the epidermis region [55]. Since this disease involves the concurrent activity of multiple cytokines, TNF-α works best when combined with IL-17A and IL-17C [30,56]. The combination produces a preferable synergistic induction to generate chains of T cell expression [12]. For instance, TNF-α steadily integrates with IL-17A mRNA to upregulate IL-17A signaling and simultaneously initiates the overexpression of IL-17R on keratinocytes, causing them to become hyperproliferative [30,57]. TNF-α can also trigger DCs to release IL-23, stimulating Th17 cells. Concomitantly, keratinocytes are triggered by both TNF-α and cytokines from stimulated Th17 cells, causing them to undergo proliferation and produce chemokines [58].

**Figure 2:**
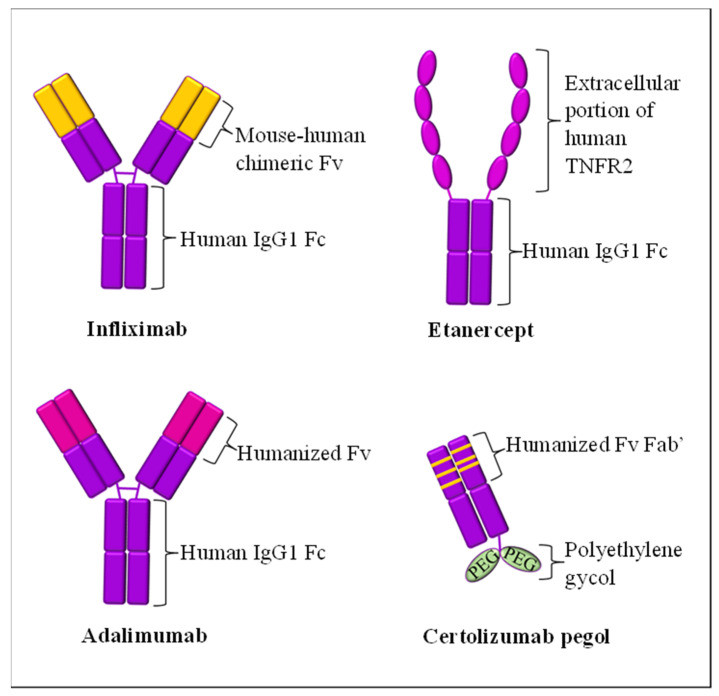
TNF-α inhibitors. Infliximab and adalimumab depict almost similar molecular structures with different Fv regions. Etanercept has specialized extracellular portions of human TNFR2. Certolizumab pegol is the most unique, as its Fc region is replaced with PEG molecules to lengthen its half-life.

In conjunction, anti-TNF-α biologics, such as etanercept, infliximab, adalimumab and golimumab, as illustrated in Figure 2, function to target and prevent the TNF-α in the inflammatory environment from stimulating other immune components. The function of TNF-α inhibitors differs from their respective molecular structure [59]. For example, etanercept is a type of psoriasis biologic that is made up of a large dimeric protein fusion of the extracellular region of TNFR2 fused with the Fc section of the humanized IgG1 mAb. It can capture the soluble and non-membrane-bound circulatory TNF-α, preventing it from binding to its receptors or TNF-α trimers, regardless of a weak binding [60,61]. It has been reported that etanercept is well tolerable in psoriasis patients, including pediatric and geriatric groups, for up to four years of treatment [62].

Meanwhile, infliximab and adalimumab share almost the same structures, but have different constituents. Infliximab is a chimeric, with 75% of it made up of a human IgG1 original section and 25% made up of a murine-derived antigen-binding variable section [63]. It is highly specific, whereby it can only neutralize the biological activity of TNF-α compared to TNF-β, although these two cytokines exhibit quite similar molecular structures [64]. Infliximab binds with a high affinity towards the circulating and transmembrane-bound TNF-α. Therefore, TNF-α will be inhibited from binding to its receptors and cellular lysis, which generates TNF-α, will also be prevented [65,66]. It can be concluded that infliximab can suppress any TNF-α-mediated connecting cascades of cellular proliferation and programmed cell death [67]. A collective of evidence stated that psoriasis patients who received a long-term subcutaneous injection of infliximab in their treatment regime experienced improved skin lesions and other related health conditions, such as psoriasis arthritis [68].

Although the molecular structures of adalimumab are almost similar to infliximab, it is not structurally chimeric, since it is made up of fully IgG1 human mAb, similar to golimumab [65,69]. Adalimumab is suitable for moderate-to-severe psoriasis patients in long-term treatment [70]. Its mechanism of action is also similar to infliximab. Nevertheless, infliximab is superior to adalimumab, since adalimumab is not highly specific when compared to the other two. This is due to the various binding affinities of adalimumab towards different antibody–antigen interfaces, whereby the force is lower than infliximab [65]. Blocking the free-bound and trans-membranous TNF-α in psoriasis from binding to a TNFR2 receptor can neutralize this overproduced cytokine. Hence, the downstream TNF-α signaling pathways that play a role in exacerbating psoriasis can be inhibited/blocked [51].

Meanwhile, on a side note, golimumab is better to administer in psoriatic arthritis patients instead of other psoriasis types [71]. Since golimumab has a high affinity and specificity towards TNF-α, it works well in blocking TNF-α from interacting with its receptor, hence neutralizing its downstream bioactivity. These bioactivities include neutralizing TNF-α-induced cell surface expression to block the adhesion activities of E-selectin, vascular cells and human endothelial cells [72]. To achieve 50% neutralization, golimumab requires less concentration when compared to infliximab and adalimumab, a property which is similar to etanercept. Golimumab is not exclusively used for psoriasis; however, it is the only FDA-approved psoriasis arthritis and rheumatoid arthritis drug/treatment [73].

In addition, among these listed TNF-α inhibitors, infliximab is the most efficient drug/inhibitor compared to adalimumab, golimumab and etanercept, given its high affinity and a PASI score that decreases by at least 75% after its administration [74]. Inhibiting the overproduced TNF-α in severe cases of psoriasis using different inhibitors opens many discussions relating to how different clinical efficacies can result in its improved biological activities. These drugs stated herein are only the primary example, since their usage is quite common.

Another TNF-α-blocking agent which has recently been discussed and explored is certolizumab pegol. It is an Fc-free with polyethylene glycol (PEG) biologic with chemical structures that are often described as peculiar since it has no Fc region. Due to its uniqueness, it disables the binding with the neonatal Fc receptor for IgG (FcRn) and, hence, minimizes the placental transfer from mother to the fetus [75]. Moreover, the PEG structure allows certolizumab pegol to undergo PEGylation to lengthen its half-life by up to 14 days [76]. Certolizumab pegol is not intensively discussed concerning psoriasis since it is commonly administered for rheumatoid arthritis [77]. This biologic is FDA-approved for psoriatic arthritis; hence, it is still yet to be exclusive solely for psoriasis. Nevertheless, this drug potentiates an excellent efficacy in improving psoriasis and has an acceptable safety profile [78].

### 4.2. IL-17 Inhibitors

IL-17 is also a promising inhibitory target in psoriasis. This inflammatory cytokine is generally involved in inflammatory cascades and reconstructing the outer cellular barrier. IL-17 is rather unique since it has multiple significant families which share homology properties, such as almost similar molecular structures [79,80]. To date, the most explored IL-17 additional families are IL-17A, IL-7B, IL-17C, IL-17D, IL-17E and IL-17F [81,82,83,84]. A previous study has shown that some of these cytokines undergo a prominent, differed expression in skin-manifested diseases, including psoriasis. In psoriasis, with the presence of IL-6 and transforming growth factor-β, CD4^+^ T cells will undergo differentiation into Th17. This event will lead to the secretion of proinflammatory cytokines, including IL-17 [85]. In a general understanding pertaining to psoriasis, IL-17 leads to the elevated expression of proinflammatory factors as well as promoting NF-κB and mitogen-activated protein kinase (MAPK) pathways [86]. IL-17 is also the key mediator in neovascularization, endothelial irregularities and coagulation, which leads to thrombosis, as well as arterial hypertension. This is one of the reasons why psoriasis is often correlated with cardiac dysfunctionality [87,88].

Among these listed additional families, IL-17A stands out the most as playing the central role in autoimmune diseases, followed by IL-17C and IL-17F [89,90]. By referring and comparing them to IL-17A in terms of the proteomic degree of conservation, we find that IL-17F has the most similarities at 55%, while IL-17E depicts the least similarities at 16% [91]. Nevertheless, in regard to autoimmunity and chronic inflammatory diseases, IL-17A, IL-17C and IL-17F act as the key mediators in manipulating the pathway cellular machinery [79,90,92,93].

In psoriasis, IL-17A directly enhances keratinocyte gene expression, such as that of cathelicidin (LL-37) antimicrobial peptides [94], via its target receptors, IL-17RA and IL-17RC [95]. During the early onset of psoriasis, LL-37 initiates the immune pathway, which eventually activates T cell-expressing IL-17A subsets, such as Th17 cells. These Th17 cells release several cytokines, including IL-17A and IL-17F, as the inflammatory biomarkers to quickly render keratinocyte hyperproliferative [96,97]. The released IL-17A further aggregates neutrophils and simultaneously inhibits the apoptosis mechanism of neutrophils and elicits neovascularization by assisting the angiogenesis pathway [98,99]. Since IL-17A has a rather stabilized mRNA, it can also work synergistically with TNF-α to exacerbate cytokine storming and the overexpression of keratinocytes [30,100]. The investigation of serum IL-17A in psoriasis patients revealed the increment of IL-17A, indicating a more highly lesioned microenvironment [101,102,103,104,105].

IL-17A is the pillar foundation of other additional IL-17 families as it is the main key effector cytokine in regard to IL-17. IL-17F shares half of its homologous form with IL-17A, although IL-17A is thought to be more potent than IL-17F [95,106]. The latest findings relating to IL-17F in psoriasis depict a quite ambiguous role, but show that it still may be the effector of IL-6 production, which is the pro-inflammatory cytokine entailing inflammation [107]. IL-17F-altered expression leads to intensified psoriatic skin inflammation, as proven in both preclinical and clinical findings [19,108,109,110].

On another note, IL-17C, which is a rather more newly discovered IL-17 family mostly affecting keratinocytes, is 23% homologous with IL-17A and can connect to its own [80,110]. Structurally, the composition of the IL-17C receptor includes IL-17RE, which has the highest level of specificity of those specialized to IL-17C [56]. In a preclinical investigation of psoriasis, IL-17C was found to be highly upregulated and was suspected to largely contribute to the formation of psoriatic dermatitis in mice [104]. Some clinical findings suggested that IL-17C intensifies the plaque formation on psoriatic skin biopsies, whereby IL-17C is overexpressed up to 125 times more than IL-17A [56,110,111,112]. Herein, the discussion is focused on IL-17A, IL-17C and IL-17F, since these families mediate autoimmunity the best, albeit in the presence of other additional families. In addition to IL-17A being the main driver in mediating inflammation, this cytokine is locally overproduced at the psoriatic lesioned area; hence, tackling it in order to neutralize or stop its overproduction is a great idea to ameliorate painful flaring patches [113]. This discovery opens up numerous discussions on the inhibition of IL-17 as one of the strategies for alleviating psoriasis inflammation with biologic drugs.

Secukinumab is a type of biologic drug, approved by the FDA in 2015, constructed from a recombinant human mAb of IgG1, and which can selectively bind to IL-17A and IL-17F [114]. This drug is suitable to treat moderate-to-severe plaque psoriasis, hypertrophic palmoplantar psoriasis, generalized pustular psoriasis and active psoriatic arthritis in adult patients [115]. When all conditions (no hypersensitivity contracted, pass the tuberculosis initial evaluation and not taking live vaccine injections) are met, secukinumab serves as the first-line biologic therapy when patients experience intolerability and are approaching the possibility of multi-organ failure after systemic therapy administration [116]. The mechanism of action of secukinumab in psoriasis focuses on targeting the IL-17A released from Th17 to block it from binding with IL-17R. Thus, antimicrobial peptides and the subsequently released cytokines, such as IL-17A and IL-17F, can also be reduced and blocked [117]. The successful blocking of IL-17A from proceeding with the signaling cascade leads to benefits, such as lowering keratinocyte hyperproliferation, preventing T cells infiltration and halting the overexpression of pathogenic genes [116]. The administration of secukinumab has shown success in some large randomized, double-blind and placebo-controlled trials. Patients receiving this biologic give positive clinical feedback, especially in relation to relieving palmoplantar and nail psoriasis, as well as plaque psoriasis. Despite potential drawbacks, such as headaches and upper respiratory tract infections, which are are inevitable, psoriatic patients are mostly well adapted to secukinumab administration [114].

Meanwhile, ixekizumab is another IL-17 inhibitory biologic drug. It is a humanized mAb IgG4 with a high affinity to specifically bind to IL-17A [118]. It has been approved by the FDA in 2016 for the treatment of moderate-to-severe plaque psoriasis in adults, and has recently been used as a treatment for psoriatic arthritis as well [119]. The mechanism of action of ixekizumab is similar to that of secukinumab, which involves targeting IL-17A in psoriasis pathogenesis. Some studies have suggested that ixekizumab is more efficient than etanercept in downregulating cytokine chaos in psoriasis after just two weeks of administration [94,120]. Indeed, the quality of life in psoriatic patients receiving ixekizumab has been reported to be positive after a good assessment on the Dermatology Life Quality Index (DLQI) simultaneously with an improved PASI score, including the clearing of lesions within one year of the study being conducted [121].

Another IL-17 inhibitor joining the mainstream biologic treatment strategy of psoriasis is brodalumab. Brodalumab is a recombinant, fully humanized mAb IgG2 that was approved by the FDA in 2017 for the treatment of psoriasis vulgaris and pustular psoriasis [122]. This biologic is an interesting one, since it is the first IL-17 inhibitor drug that blocks and neutralizes IL-17 receptors due to its high affinity instead of the IL-17 cytokine itself [123]. After administration, brodalumab binds to IL-17A and IL-17C receptors, IL-17RA and IL-17RC. This action concomitantly blocks IL-17 from binding, thus downregulating psoriatic inflammation, such as lesioned skin transcriptome, and neutralizes associated gene expression. Hence, chemokine and IL-23 production are halted to further reduce psoriatic inflammation [124,125]. Such an impactful mechanism of action in blocking IL-17 by blocking IL-17 inhibitors brings benefits to psoriasis patients, including reduced inflammation of lesions and direct improvement in cardiovascular diseases [121,126].

### 4.3. IL-23 Inhibitors

The IL-23 structure has two main subunits, which are p19 and p40, which are specific to IL-23 and IL-12/IL-23, respectively. IL-23 is primarily produced by DCs and macrophages, while its receptors are commonly expressed on T cells, NK cells, neutrophils, macrophages and mast cells. With the presence of TNF-α, IFN-γ and other transcription factors, IL-23 can further enhance its regulation via the TLR signaling pathway [127]. In terms of its mechanism of action, IL-23 firstly binds to its receptor to form an IL-23/IL-23R complex. This can initiate Th17, Th22, CD4^+^, CD8^+^ and γδ T cells to produce pro-inflammatory cytokines, such as IL-17 and IL-22, which play roles in inflammatory pathways and neovascularization [124,128]. If IL-17A is absent in the event, IL-23 will stimulate keratinocytes, making them become hyperproliferative. Other mechanisms of IL-23 include triggering the proliferation of macrophages to generate more TNF-α and amplifying IL-23R expression to form a self-amplifying loop [128,129]. These events, in which IL-23 is overexpressed, are devastating in immune-mediated diseases, such as psoriasis, especially since there is evidence of upregulated IL-23 in psoriatic lesions [128,130]. Therefore, IL-23 does play a significant role in the pathogenesis of psoriasis from the early onset to its sustenance mechanisms [122,131]. The advanced technology of using biologics in inhibiting IL-23 in psoriasis has succeeded, giving promising results [132].

Tildrakizumab is a fully human mAb of IgG1 kappa suitable for adults who have moderate-to-severe psoriasis. It was approved by the FDA in 2018 and has a high affinity for selecting the p19 subunit of IL-23 [133]. Due to this characteristic, tildrakizumab will bind to this subunit, causing the later event of cytokine signaling to be prohibited. Hence, other downstream pro-inflammatory mediators will also be halted [134]. Tildrakizumab is more effective than TNF-α inhibitors, such as etanercept, as it is proven to achieve PASI 75 on the 12th week of administration [135].

Tildrakizumab’s mechanism of action is similar to other IL-23 inhibitors, such as guselkumab and risankizumab. Both biologics are a fully human mAb of IgG1, approved by the FDA for moderate-to-severe plaque psoriasis in adult patients [122,136]. These drugs are considered in mainstream biologics, since they are proven to lower the overexpressed IL-6, IL-17A, IL-17F and IL-22 as early as four weeks before the administration. This is concurrent with the reduced discomfort signs and symptoms of psoriasis as well [137].

### 4.4. IL-12/23 Inhibitors

IL-12 belongs to the heterodimeric cytokine IL-6 superfamily and has a structural β-chain subunit of p40 similar to IL-23; the only difference is in its α-chain, which commonly comprises a p35 instead [138]. Commonly, IL-12 is produced by DCs, macrophages, monocytes and B cells. In terms of biological activities, IL-12 and IL-23 primarily contribute to the expansion of Th1 and Th17 cells, respectively. In psoriasis, the common p40 subunit is the succeeding key that binds to its receptor to form its complex formation. Early investigations in both preclinical and clinical studies have shown that the p40 subunit of these pro-inflammatory cytokines is overexpressed [129,139]. This is necessary for the subsequent immunoregulation for recruiting pro-inflammatory Th1 and Th17 cells and triggering the release of their associated pro-stimulatory cytokines [140].

Most CD4^+^ T cells express IL-12 and its subunits, such as IL-12Rβ2 and IL-12p40, which further initiate the differentiation of T cells via Toll-like receptor signaling. This event will consequently overproduce IFN-γ [141]. Meanwhile, IL-23 can bind to IL-23R to initiate the secretion of multiple cytokines, such as IL-17, IL-22, IL-26, IFN-γ and TNF-α, as well as CCL20 [142]. Through the combination of the signal transducer and activator of transcription (STAT)3 and RAR-related orphan receptor gamma (RORγt) working together, IL-23 and IL-23R can transactivate to harmonize a positive feed-forward loop to reinforce IL-23R, IL-17 and IL-22 expressions. Eventually, the Th17 phenotype can achieve its stabilization, which is required for sustaining cytokine secretion, including IL-17 for keratinocyte hyperproliferation [142,143]. All these events are aggravated by the p40 subunit of both IL-12 and IL-23. Thus, p40 serves as the most favored target to inhibit the downregulation of the overall biological activities of IL-12 and IL-23, especially in psoriasis [140,142,143].

**Figure 3:**
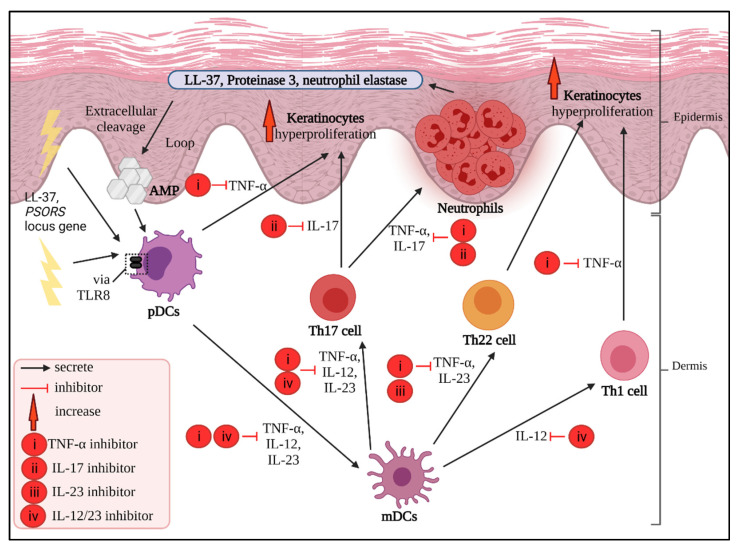
Cytokine inhibitors and their respective blocking interests based on the postulated psoriasis pathogenesis. The overexpressed cytokines released by respective cells can be blocked, and this can downregulate the further inflammatory pathway.

Ustekinumab is the only sole prime IL-12/23 inhibitor to date. It is a humanized mAb IgG1κ, and can bind to the p40 subunit of both IL-12 and IL-23 and disturb the downstream immunoregulation. It is suitable to administer to moderate-to-severe psoriasis patients due to its efficacy profiling [140,144]. The efficacy of ustekinumab in psoriasis can be seen by comparing mRNA expression alteration using microarray analysis. Concurrent with more than 75% PASI score amelioration, this IL-12/23p40 inhibitor caused a significant difference in approximately 5000 of the genes modulated. As a result, TNF-α is suppressed due to the successful blocking of IL-12/23, which provides subsequent IL-17-associated gene downregulation. In comparison with etanercept, ustekinumab excels in suppressing multivariate psoriasis-associated genes and cytokines such as IL-1, IL-22, IFN-γ and IL-17. Although it is unfortunate to learn that ustekinumab does not have enough data to support long-term usage, this dual-functioning inhibitor is proven to enhance the quality of life in psoriatic patients [145,146,147]. The cytokine inhibitors used in psoriasis treatment are summarized in Table 1, and the simplified mode of action of all inhibitors are illustrated in Figure 3.

## 5. Other Pro-Inflammatory Cytokines Candidates

From the understanding of the stated cytokines, which provides excellent ideas and a framework for establishing biologics, perhaps other cytokines may render similar benefits in alleviating psoriasis. In immune-mediated inflammatory diseases, pro-inflammatory cytokines are being produced to create an exacerbated inflammatory environment. Herein, other potential cytokines are briefly described in relation to their association with psoriasis and insights into the advantages of targeting them as a treatment plan.

The IFN group is proposed to be beneficial if it is targeted during the early onset of psoriasis progression, especially for paradoxical psoriasis. Type I IFN (IFN-α and -β) is mainly secreted by pDCs and keratinocytes, which are responsible for recognizing psoriasis autoantigens, such as LL-37 peptides, through TLR-7. It helps in pDC maturation and helps T cells to secrete cytokines such as IL-22, which is necessary for STAT3 phosphorylation and keratinocyte rapid division [147]. Plasmacytoid DC infiltration during this preliminary stage is inevitable, as it is an early response to the skin injury and/or due to the presence of autoantigens. The secretion of Type I IFN can be sustained to further progress the psoriatic inflammation until it enters the adaptive immune response activation phase. Thus, this explains the reason why Type I IFN is absent in chronic psoriatic lesions [148,149]. In regard to sustained Type I IFN production, it has also been postulated that blocking TNF may prolong Type I IFN production [150]. Blocking Type I IFNs may inhibit T cells from continuing to develop psoriasis, as reported in an in vivo experiment by Gui and team [151]. Since the presence of this cytokine is significant in the pre-psoriasis microenvironment, perhaps future research may innovate some topical treatments, revised phototherapy regimes or even biologics that can inhibit localized Type I IFN, hence preventing downstream inflammatory regulation.

Similarly, the high level of Type 2 IFN also aids T cells in migration towards the inflammation site, and hypothetically acts as an important linker between inflammatory T cells and keratinocytes [152]. Earlier data suggested that all clinical types of psoriasis, namely, plague, erythrodermic and guttate, concluded a significant positive correlation between PASI score and high levels of IFN-γ [153]. The role of IFN-γ in psoriasis vulgaris remains to be discovered, since there is no relationship between highly elevated IFN-γ with both the mean values of IFN-γ and PASI score [154]. Other previous findings are in an agreement with this different pattern; elevated IFN-γ may or may not correlate with the PASI score [155]. The disparity of findings suggested that IFN-γ may play a role in psoriasis pathogenesis, but this is somewhat ambiguous; therefore, it is not considered to be the definite regulator or a single player in the overall pathways [153,154]. Nevertheless, it still contributes to the psoriasis cytokine storm; hence, other postulations suggested that IFN-γ is synergic with TNF-α. Using transcriptome analysis, these cytokines are found to be a part of a similar process in psoriasis and atherosclerosis when it comes to mediating inflammation. Both cytokines are elevated in moderate-to-severe psoriasis and atherosclerotic plague formation [156]. This idea brings us back to the previous statement that psoriasis is closely related to cardiovascular disease as its comorbidity [87,88]. Therefore, instead of IFN-γ alone, future research may want to further explore this IFN-γ/TNF-α synergism, which possibly can establish an ultimate inhibitor for both cytokines at one time.

In IL-1, especially the IL-1β cytokine family, which is secreted by macrophages, DCs and keratinocytes are known to play a role in mediating psoriatic inflammatory pathways, including the sustained production of LL-37 peptides [157,158]. This cytokine can initiate the productivity of IL-17 for γδ T cell differentiation and activation and the further secretion of chemokines. In psoriasis, the significant elevation of IL-1β mRNA and its protein expression level has been observed in the affected skin of psoriasis patients [159]. The same elevation pattern is also observed for IL-36, the IL-1 subfamily in psoriasis [160]. It is known that IL-36 is one of the factors to produce LL-37 peptides by stimulating keratinocytes and activating antigen-presenting cells [161]. IL-36 is also the mediator in angiogenesis and forming Munro’s microabscesses. Macrophages that are stimulated by IL-36 can contribute to the increased levels of IL-23 and TNF-α [162]. With all the data gathered, targeting IL-1 and its subfamily, IL-36, in psoriasis can potentially halt inflammatory pathways intracellularly.

IL-6 is one of the most significant cytokines to be intensively elevated by DCs, Th17 cells and even keratinocytes during psoriasis flare, since it plays a major role in mediating inflammation. With the help of IL-6, cells such as DCs, macrophages and keratinocytes can expand their growth and, hence, increase the production of other cytokines. IL-6 is also required in the differentiation phase of Th17 cells and for endothelial cells to express their adhesion parts. IL-6 can be the targeted target as well, since it engages in some cross-talks with the IL-23/Th17 axis, which is thought to have a substantial role in psoriatic inflammation [163]. Targeting IL-6 has led to promising results in slowing down these uncontrolled biological activities in exacerbating psoriatic lesions, and even in reducing atherosclerosis risk comorbidity [164].

In psoriasis, elevated levels of IL-8 are known to promote keratinocytes to exponentially grow in multiple layers and stimulate neutrophil aggregation. Angiogenesis can be even more advanced with the help of IL-8, thus explaining why psoriasis patients have a greater risk of cardiovascular comorbidity [10,165,166]. Targeting the increasing abundance of IL-8 in psoriasis patients may help to prevent these events from happening [167].

The same strategy of halting the elevated pro-inflammatory cytokine in psoriasis may work with IL-21, which is majorly secreted by CD4^+^ T cells, Th17 cells and T follicular helper cells [168]. The elevated overall IL-21 secreted by CD4^+^ T cells and Th17 cells in the serum of psoriasis patients is significantly correlated with a progressive PASI score. In the same experiment, IL-21 is proven to mediate the differentiation of CD4^+^ T cells to Th17 cells, which has been shown by other similar findings, in addition to the downregulation of Treg cells [169,170]. Moreover, IL-21 induces the proliferation of matured B cells by regulating its class-switching process and helps to increase IL-21 and IL-17A secretion [170,171]. These events can be avoided if IL-21 is blocked, and hence downregulates the excessive productivity of Th17 cells. In regard to said Th17 cells, these cells, aided by transcription factor retinoid-related orphan receptor (ROR)-γt and STAT3 and IL-23 secrete IL-17A, IL-22 and TNF-α (Th17 cytokines) and IL-17 for keratinocyte hyperproliferation [172,173,174]. The correlation of these stated interleukins has been heavily proposed, and research shows that IL-17 works remarkably well in tandem with IL-23, creating an axis called the IL-17/IL-23 axis pathway [26]. In an intensive review by Liu and team [174], the IL-17/IL-23 axis is found to be the next possible candidate for downregulating skin inflammatory diseases, such as psoriasis. IL-23 functions to promote the proliferation of CD4^+^ T cells into Th1 cells and Th17 cells. Perhaps future research can investigate in depth the relationship between IL-17 and IL-23 to innovate a binary inhibitor that can halt both cytokines simultaneously.

IL-22 is a rather interesting one, considering that it is a pleiotropic interleukin in which its pro-inflammatory properties may outdo its anti-inflammatory properties when in an excessive amount. In a healthy condition, the IL-22 expressed by keratinocytes is beneficial for wound healing and tissue repair [175]. During psoriatic flares, cells such as lesional T cells secrete IL-22 for epidermal hyperplasia, but do not engage with keratinocyte proliferation directly [176,177]. In psoriatic pediatric patients, IL-22 is found to be at a higher level compared to adult patients, showing that its pathogenesis may slightly differ from adults [6,178]. In later evidence, IL-22 triggers Th17 cells, which are required for keratinocyte divisions [179]. This finding correlates with the presence of elevated IL22 gene promoters during the early age of psoriasis onset [180]. However, IL-22 is still responsible for upregulating psoriasis mostly by collaborating with other immune components. In attempting to suppress IL-22, future research potentially can take an approach of targeting its soluble scavenging receptors instead, which mostly includes the IL-22-binding protein IL-22BP and/or IL-22RA2. Focusing on IL-22BP, it has been proven, in an in vivo experiment conducted by Voglis and team [181], that this structure is the mediator in propagating psoriasis inflammation, suggesting that other autoimmune diseases may exhibit a similar process. Using this information, IL-22BP may be one of the best candidates to be inhibited in psoriasis.

The idea of blocking these cytokines as summarized in Table 2 must be aligned with the possible limitations and/or adverse effects, which may worsen psoriasis and create other conditions. To date, the possible risks of these inhibitions are rather inconclusive and incomplete, since most of the clinical trials are still ongoing, and some have even come to termination. The absence of a certain kind of cytokine may be compensated by other cytokines, which may create alternated biological events or idiopathic comorbidities. Blocking dual-functioning and synergistic cytokines may need more data, trials and insights into the overall extended eventuality. It is very important to consider all the potential outcomes, considering that administered inhibitors will be circulating throughout the body and not exclusively on psoriasis lesions only.

## 6. Conclusions

The main potential cytokines listed in this review are the most significantly targeted cytokines that have shown effectiveness in alleviating psoriasis, as previously reported. A rather wide point of view of complex psoriasis pathogenesis must be mapped by connecting each piece of information, which consequently can help to avoid any paradoxical conclusions. By analyzing this gathered information on psoriasis, the authors hope to inspire future researchers to establish new treatment ideas and continue to explore the myriad of cytokine profiling with their numerous biological interactions, even if this is considered to be minute detail. Although cytokine inhibitors seem promising, the data findings still leave some research gaps, especially in terms of long-term impacts, vast globalized clinical data, dosage concern, risks assessment and likely multiple adverse effects. Since these biologics are not personalized, more targets of interest must be experimented with and, hence, encourage the researcher to investigate other potential cytokines as well as their biosimilars. Attempting new strategies for establishing new inhibitors must be appropriate with individual cytokine profiling and heavily based on clinical evidence, such as the elevation of serum inspection and PASI scores.

By understanding the fundamental mechanisms of action of each potential cytokine in psoriasis, perhaps the issues, including cytokine compensation drawbacks, dual recognition and synergism, can be tackled, leading to the innovation of more advanced biologic treatments with the minimum side effects possible. Every trial further demonstrates how beneficial it is to inhibit these stated cytokines, and perhaps more novel bioactivities can be discovered in regard to psoriasis. Blocking these cytokines may contribute knowledge for treating refractory psoriasis patients, especially classical psoriasis conditions, simultaneously with an understanding of the inhibitors that alter the relationship with other immune components.

## Figures and Tables

**Table 1:** Summary of cytokine inhibitors used in psoriasis treatment.

Cytokine Targets	Biologic Drug Name (Brand)	Year of FDA Approval for Psoriasis Treatment	Molecular Structure	Mode of Action	Possible Side Effects	References
TNF-α	Infliximab (Remicade^®^)	2006	Human-mouse chimeric combination of mAb IgG1	Inhibit circulating and transmembrane-bound TNF-α	Upper respiratory tract infection, hepatotoxicity, tuberculosis risk, worsening psoriasis	[63,65,66]
	Etanercept (Enbrel^®^)	2004	Extracellular region of TNFR2 fusion with humanized mAb IgG1	Inhibit soluble and non-membrane-bound circulatory TNF-α from binding to TNFR2 receptor	Upper and lower respiratory tract infections, rhinitis, pharyngitis, tuberculosis risk	[60,61]
	Adalimumab (Humira^®^)	2008	Humanized mAb IgG1	Inhibit circulating and transmembrane-bound TNF-α	Upper respiratory tract infection, sinusitis, urinary tract infection	[64,69]
	Golimumab (Simponi^®^)	Not applicable *	Humanized mAb IgG1κ	Inhibit circulating and transmembrane-bound TNF-α	Recurring psoriasis flare	[72]
	Certolizumab pegol (Cimzia^®^)	Not applicable *	Humanized Fab subunit to mAb fusion, with Fc-free PEGylation and no Fc region	Inhibit circulating and transmembrane-bound TNF-α	Urinary tract infections, gastroenteritis, nasopharyngitis, headache, pruritus, tuberculosis risk	[75]
IL-17	Secukinumab (Cosentyx^®^)	2015	Humanized mAb IgG1	Inhibit IL-17A and IL-17F	Nasopharyngitis, diarrhea, mucocutaneous candidiasis, upper respiratory tract infection, neutropenia	[114,117]
	Ixekizumab (Taltz^®^)	2016	Humanized mAb IgG4	Inhibit IL-17A	Candidiasis, irritable bowel syndrome, neutropenia	[118]
	Brodalumab (Siliq^®^)	2017	Humanized mAb IgG2	Block IL-17A and IL-17C receptors	Arthralgia, headaches, fatigue	[122,124,125]
IL-23	Tildrakizumab (Ilumya^®^)	2018	Humanized mAb IgG1κ	Inhibit IL-23 alpha subunit; p19 subunit	Inflammatory bowel syndrome, acute myocardial infarction	[122,136]
	Guselkumab (Tremfya^®^)	2017	Humanized mAb IgG1λ	Inhibit IL-23 alpha subunit; p19 subunit	Upper respiratory tract, nasopharyngitis, headaches, infection	[122,136]
	Risankizumab (Skyrizi^®^)	2019	Humanized mAb IgG1	Inhibit IL-23A	Nasopharyngitis, upper respiratory tract infection, headache, arthralgia, back pain, diarrhea	[122,136]
IL-12/23	Ustekinumab (Stelara^®^)	2009	Humanized mAb IgG1	Simultaneously inhibit p40 subunit of IL-12 and IL-23	Tuberculosis risk	[140,145]

* FDA approved for psoriasis arthritis only.

**Table 2:** Potential cytokines of interest to be targeted in psoriasis.

Cytokines Target	Mode of Action in Psoriasis	Expected Biological Inhibitory Activities in Psoriasis	Expected Side Effects	References
Type I IFN (-α, -β)	Induce T cells to produce IL-22 for keratinocyte proliferation. Mediate CD8^+^ T cells to infiltrate the dermal area. Promote B cells to differentiate into antibody-secreting plasma cells. Activate myeloid dermal DCs to upregulate co-stimulatory substances and HLA molecules. Stimulate cDCs and DCs to secrete IL-23 for Th/Tc17 polarization.	Blocking pDC and cDC maturation. No induction of other autoimmune cells for chronic-relapsing pathogenic condition. Reduce the initial inflammatory pathway.	Not yet documented	[47,147,182,183,184,185]
Type II IFN (-γ)	Promote keratinocyte proliferation by inducing the BCLx protein and altering other antiapoptotic factors. Establish intercellular adhesion molecule 1 (ICAM-1) and HLA-DR expressions for immunoregulatory of T cells.	Reduce the production of pro-inflammatory cytokines and mediators. Reduce lymphocytes from circulation to migrate at the inflammation site.	Not yet documented	[154,182,183]
IL-1β	Stimulate IL-17 for γδ T cell proliferation for chemokine secretion.	Reduce IL-6 and IL-8 production necessary for angiogenesis.	Recurring psoriasis flare	[159,182,183]
IL-36	Produce LL-37 peptides by stimulating keratinocytes and activating antigen-presenting cells. Angiogenesis mediator	Suppress macrophages to secrete IL-23 and TNF-α. Reduce the formation of Munro’s microabscesses.	Not yet documented	[161,182]
IL-6	Promote Th17 cells and cause endothelial cells to differentiate. Angiogenesis mediator. Stimulate DCs, macrophages and keratinocytes to secrete cytokines.	Disturb the early initiation of immune cells interaction. Downregulate angiogenesis. Suppress IL-23 secretion by DCs, macrophages and keratinocytes.	Inducing psoriasis onset	[30,161,182]
IL-8	Keratinocyte hyperproliferation. Promote neutrophil aggregation. Angiogenesis mediator.	Reduce the formation of Munro microabscesses. Reduce the risk of cardiovascular conditions related to psoriasis.	Irritation, pain, itch, edema	[10,161,167,182]
IL-21	Promote CD4^+^ T cells to differentiate to Th17 cells. Promote B cell proliferation. Cause population imbalance of Th17 and Treg cells.	Reduce the key factor for Th17 formation. Restore the balance of Th17/Treg cells to proportion.	Not yet documented	[169,170,171,182]
IL-17/IL-23 combination	Increase the production of Th17 cytokines. Promote inflammatory signaling pathways. Keratinocyte hyperproliferation.	Restrain the positive feedback loop of secreted Th17 cytokines. Downregulate keratinocyte hyperproliferation.	Not yet documented	[86,174,182]
IL-22	Increase epidermal hyperplasia. Mediate Th17 cells for keratinocyte hyperproliferation.	Downregulate Th17 cells for hyperproliferation.	Not yet documented	[176,177,182]

## Data Availability

Not applicable.
